# MYC and AMPK–Save Energy or Die!

**DOI:** 10.3389/fcell.2017.00038

**Published:** 2017-04-11

**Authors:** Heidi M. Haikala, Johanna M. Anttila, Juha Klefström

**Affiliations:** Research Programs Unit/Translational Cancer Biology, Cancer Cell Circuitry Laboratory, Institute of Biomedicine, University of HelsinkiHelsinki, Finland

**Keywords:** MYC, AMPK, cancer metabolism, apoptosis, glycolysis, glutamine metabolism, anaplerosis, synthetic lethality

## Abstract

MYC sustains non-stop proliferation by altering metabolic machinery to support growth of cell mass. As part of the metabolic transformation MYC promotes lipid, nucleotide and protein synthesis by hijacking citric acid cycle to serve biosynthetic processes, which simultaneously exhausts ATP production. This leads to the activation of cellular energy sensing protein, AMP-activated protein kinase (AMPK). Cells with normal growth control can stop cell proliferation machinery to replenish ATP reservoirs whereas MYC prevents such break by blocking the cell cycle exit. The relentless cell cycle activation, accompanied by sustained metabolic stress and AMPK activity, switches the energy-saving AMPK to pro-apoptotic AMPK. The AMPK-involving metabolic side of MYC apoptosis may provide novel avenues for therapeutic development. Here we first review the role of anabolic MYC and catabolic AMPK pathways in context of cancer and then discuss how the concomitant activity of both pathways in tumor cells may result in targetable synthetic lethal vulnerabilities.

## MYC–exploiting vulnerabilities in cancer metabolic programs

The classical view of oncogenic MYC expression being a cell cycle reprogrammer has recently broadened in the light of new genome-scale promoter and transcriptomic studies, which have exposed MYC's widespread transcriptional impact across the genome and especially on the genes orchestrating anabolic metabolism (Eilers and Eisenman, 2008; Dang, 2012; Kress et al., 2015). MYC not only directly stimulates the core cell cycle machinery, but also prepares the cells for cell division by globally stimulating cell growth and acquisition of macromolecules so that the cells can successfully progress through different cell cycle checkpoints to complete the mitotic cycle (Dang, 2013; Kress et al., 2015). Unlike healthy cells, most tumor cells cannot switch off MYC expression in response to anti-proliferative signals from outside of the cell. Hence, sustained high level MYC expression establishes an irreversible metabolic transformation, which can operate as an autonomous cell cycle machinery on its own right—via incessant generation of biomass for growth, which then consequently pushes the cell cycle progression forward (Stine et al., 2015).

This increased understanding of MYC's role in control of metabolic machinery has conceived new ideas and concepts for rational design of therapeutic synthetic lethal strategies to treat cancer. The “indirect” MYC targeting strategies are often based on the simple idea that MYC-transformed cells, since unable to exit from the cell cycle, would be extremely vulnerable to treatment that perturbs the cell growth supporting anabolic programs or limiting metabolites. Such disturbance in enforced anabolic metabolism leads to metabolic stress and (re-)activation of cell cycle checkpoints and consequently, selective induction of apoptosis in transformed cells (Stine et al., 2015). A good example of targetable MYC-dependent metabolic alteration is the striking addiction of MYC-transformed cells to availability of glutamine-derived carbon (see below). The metabolic alterations caused by MYC also stimulate AMPK activity, which can unleash both survival and apoptosis pathways (see below). While the context-dependency parameters here are still poorly understood, it is noteworthy that AMPK activity is targetable with safe drugs traditionally used for treatment of metabolic disorders. Therefore, uncovering the secrets of the pro-apoptotic AMPK function is likely to be a highly rewarding task with ample of repurpose-able drug candidates available for proof-of-mechanism testing.

Solid demonstration of the clinical feasibility of any MYC-based synthetic lethal strategy still awaits to come forth but it is important to note that at general level, the concept of attacking cancer metabolic vulnerabilities has been fully validated in the clinic. For example, treatment of cancer with chemotherapy agent fluorouracil (5-FU), which is an inhibitor of thymidylate synthase, or with methotrexate, an inhibitor of dihydrofolate reductase and folates, leads to depletion of deoxyribonucleotides and perturbed DNA synthesis, which amounts a lethal level metabolic stress to cancer cells (Longley et al., 2003).

## MYC–master of cell cycle and growth

MYC has wide variety of functions but one that stands out in nearly all experimental systems and models is a positive regulation of cell proliferation and growth. Already early findings demonstrated MYC upregulation at cell cycle entry, association of high MYC expression with proliferation active embryonic and adult tissues, as well as revealed the ability of an enforced MYC expression to induce growth factor-independent cell cycle entry and prevent the cell cycle exit (Eilers et al., 1991; Evan et al., 1992; Pelengaris et al., 2002). These observations guided many early studies to specifically focus on MYC's role in transcriptional regulation of core cell cycle and DNA replication related genes, as MYC logically was pictured as a key driver of the cell cycle machinery. MYC indeed directly regulates number of genes important for the core cell cycle machinery. For example, MYC represses the expression of CDK inhibitors p21^CIP1^, p15^INK4B^, and p27^KIP1^ (reviewed in Kress et al., 2015) and transcriptionally activates the genes of cyclin D and cyclin-dependent kinase 4 (Stine et al., 2015). MYC promotes the expression of E2F transcription factors, which mediate progression into S phase and the combined MYC and E2F activity induces DNA replication genes to both initiate and sustain DNA replication (Zeller et al., 2003, 2006; Dong et al., 2014). MYC also transcriptionally regulates miRNA cluster miR-17-92 to attenuate E2F1 functions in S-phase, which mechanism appears to be important for keeping the rate of DNA replication in check (Dominguez-Sola et al., 2007; Aguda et al., 2008). Notably, MYC localizes to early sites of DNA replication and binds many components of the pre-replicative complex, suggesting transcription-independent regulatory functions in initiation of DNA replication (Dominguez-Sola et al., 2007).

**Figure 1:**
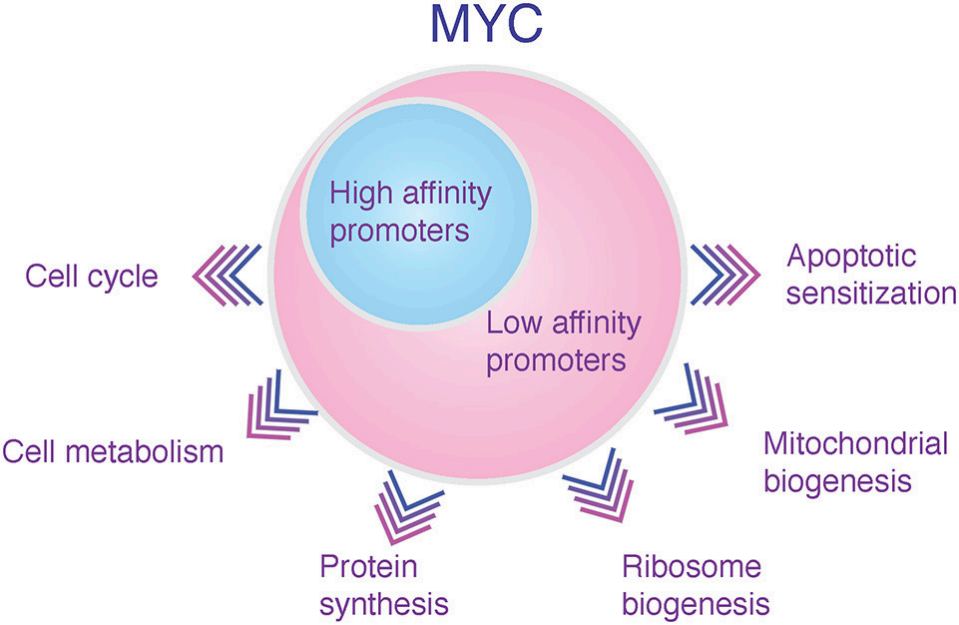
**Transcriptional domains of low and high level of MYC expression**. In the nucleus MYC binds together with MAX to E-box-containing DNA sequences when they are accessible in chromatin, occupying enhancer and promoter regions of thousands of genes. In humans, MYC binds up to 10–15% of genomic loci (Eilers and Eisenman, 2008). The supraphysiologically expressed (oncogenic) MYC targets virtually all active promoters and enhancers in the genome, postulating a role for MYC at least in these circumstances as a universal amplifier of expressed genes (Lin et al., 2012; Nie et al., 2012). However, MYC binding does not always alter the gene's transcriptional activity (Dang, 2013). Furthermore, recent investigations studying the impact of increasing MYC levels to global promoter occupancy have suggested that elevated MYC levels have only minor effect on the MYC binding to classical E-box high-affinity MYC promoters—possibly because they are already employed by the physiological MYC (depicted as a blue circle in the figure). Instead, the high MYC concentration predominantly and indirectly leads to selective occupancy of sets of enhancers and promoters with normally only weak affinity to MYC (Sabo et al., 2014; Walz et al., 2014; Lorenzin et al., 2016) (depicted as a pink circle in the figure). Such promoter “invasion,” which occurs in cells with high-level MYC expression, may lead to activation or repression of novel pathways that are not influenced by the normally regulated MYC (Wiese et al., 2015). Therefore, MYC may claim its status as a major oncogene through qualitative attributes, including new interaction patterns with companion transcription factors and off-target promoter invasion on accessible sites in the chromatin, rather than only via quantitative (general amplifier) functions (Horiuchi et al., 2012; Walz et al., 2014). Examples of MYC-regulated genes in each category of cellular functions include: Cell cycle: cyclin-dependent kinases (e.g., cdk4/6) and cyclins (e.g., cyclin E). Cell metabolism: GLUT1, LDH-A, ASCT2 and SN2. Protein synthesis: Initiation factors (eIF4E, eIF4G), elongation factors (EEF1B2). Ribosome biogenesis: NPM, ribosomal RNA. Mitochondrial biogenesis: PGC-1β, NRF-1. Apoptotic sensitization: ARF, BAX, BAK.

Recent unbiased genome-wide gene expression and chromatin immunoprecipitation (ChIP) analyses combined with next-generation sequencing have indeed corroborated earlier findings by exposing plethora of MYC regulated genes with annotated functions in cell cycle regulation and DNA replication (Figure 1). However, the new data have also broadened earlier views by demonstrating, first, that physiological (~normal) and supraphysiological (~oncogenic) levels of MYC operate partially via different gene-sets since only the supraphysiological MYC binds and transcriptionally activates/represses genes whose expression is directed by enhancer/promoter regions with low affinity for MYC:MAX heterodimers; or, with low affinity for transcription repressing complexes involving MYC and for example, MIZ-1 (Walz et al., 2014; Wiese et al., 2015). Secondly, the new data from genome-wide studies of MYC's transcription factor function indicate that substantial fraction of MYC regulated genes include regulators of cell metabolism, for example “cell growth genes,” which mediate ribosome biogenesis and protein synthesis, “energy metabolism genes” involved in glycolysis, glutaminolysis and mitochondrial biogenesis as well as “anabolic genes” including genes regulating the biosynthesis of amino acids, nucleotides and lipids (Figure 1, see below). Mitochondrial biogenesis increases bioenergetic capacity and supports biosynthesis of cellular macromolecules needed for cell proliferation and growth (Morrish and Hockenbery, 2014). MYC activates key genes involved in the mitochondrial biogenesis including PGC-1β and NRF-1 (Dang, 2013).

The new data does not change our principal view on MYC. MYC is still a major driver of the cell cycle. However, it now appears that MYC drives a very sustainable program of cell proliferation by inducing sufficient production of biomass and biosynthetic building blocks for cell growth, which ensures that one cell division results in two about equal size of cells rather than two small cells.

## MYC–master of anabolic processes

### Anabolic phenotype of cancer cells

Proliferating cancer cells have fundamentally different metabolic status compared to differentiated, mainly resting adult cells; measurable changes encompass all domains of cellular metabolism, such as bioenergetics, biosynthesis and redox potential (Cairns et al., 2011). Therefore, it is said that cells on a path to cancer undergo “metabolic transformation” and in the end of the path they develop a “cancer metabolic phenotype” (DeBerardinis, 2008). The metabolic transformation of cancer cells is often viewed as a general metabolic shift from energy production to biosynthesis. Lipid metabolism provides an illustrating example: While resting cells mainly oxidize lipids for energy production, cancer cells or any growing and proliferating cell types need to boost lipogenesis to satisfy the biomaterials needs of growing cell membranes (Stine et al., 2015).

Even Warburg effect, the quintessential cancer metabolic phenotype, can be seen as a specific adaptation to anabolic metabolism. Warburg effect is the observation that cancer cells, even in aerobic conditions, shift from oxidative phosphorylation to glycolysis for ATP production (Warburg, 1956). However, the glycolysis, which takes place in the cytosol, is relatively inefficient way to produce bioenergy in comparison to mitochondrial oxidative phosphorylation (glycolysis: 2 ATPs per glucose molecule vs. 36 by mitochondrial oxidative phosphorylation) (Vander Heiden et al., 2009). Therefore, the increased energy need of growing cells for biomass production could not possibly explain the Warburg effect. From the standpoint of anabolic metabolism, glycolysis and the parallel running anabolic pentose phosphate pathway (PPP) produce NADPH, which provides the reducing equivalents for many biosynthetic reactions, such as lipid synthesis and fatty acid elongation. Furthermore, the first product of glycolysis, phosphorylated glucose (glucose-6-phosphate), lies at starting point of both glycolysis and pentose phosphate pathway, which produces in addition to NADPH, ribose for the synthesis of nucleotides, and erythrose 4-phosphate (E4P) for the synthesis of aromatic amino acids (Vander Heiden et al., 2009). Moreover, the end product of glycolysis, pyruvate-derived acetyl-CoA, feeds lipid synthesis. These examples illustrate that while the glycolytic oxidation of glucose to pyruvate is an inefficient way to produce ATP, it still produces plenty of reducing equivalents, free energy, and carbon skeletons for biosynthesis (Vander Heiden et al., 2009).

**Figure 2:**
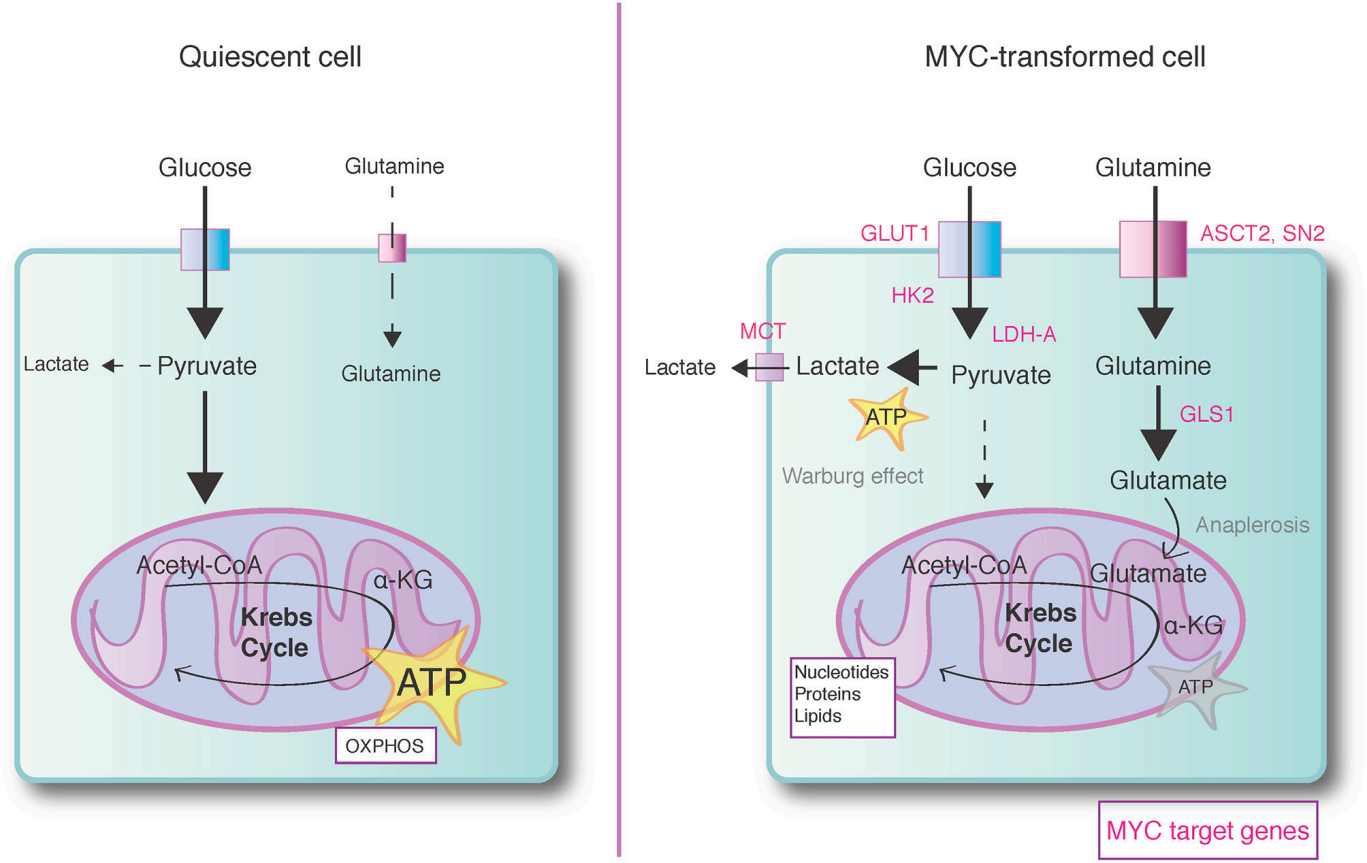
**MYC promotes anabolic metabolism**. Normal quiescent cells (*left*) predominantly rely on mitochondrial oxidative phosphorylation (OXPHOS) to generate ATP energy. Breakdown of glucose in glycolysis and mitochondrial Krebs cycle yields ATP and reducing equivalents (NADH and FADH2). The transfer of electrons from reducing equivalents to molecular oxygen during OXPHOS completes the ATP-generating processes, yielding altogether 36 ATPs per glucose molecule. Supraphysiological expression of MYC induces a shift to anabolic metabolism (*right*), which predominantly produces building blocks for biosynthesis of macromolecules (nucleic acids, proteins, carbohydrates, lipids) at cost of less energy production. While aerobic glycolysis i.e. Warburg effect produces only 4 ATPs per glucose molecule, the glycolysis and parallel running pentose phosphate pathway (not shown in the figure) generate plenty of reducing equivalents for biosynthetic reactions. MYC also enhances utilization of glutamine-derived carbon for biosynthetic reactions and MYC transformed cells may use alpha-ketoglutarate (α-KG), a product of glutaminolysis, as a key anaplerotic substrate to maintain Krebs cycle-dependent biosynthetic reactions. The metabolic target genes regulated by MYC are marked with pink color. GLUT1, Glucose transporter 1; HK2, Hexokinase 2; LDH-A, Lactate dehydrogenase A; MCT, Monocarboxylate transporter; ASCT2, ASC amino acid transporter; SN2, System N glutamine transporter 2; GLS1, Glutaminase 1.

MYC is inferred as a major player in metabolic transformation of cancer cells due to its pervasive impact on the genes encoding protein and enzyme mediators of glycolysis, glutaminolysis, mitochondrial biogenesis, and biosynthesis of macromolecules (Stine, Cairns, Kress). We single out below three metabolic pathways, which are altered by MYC and which represent the metabolic phenotype of many types of cancer cells (Figure 2).

### Glycolysis

MYC enhances glucose uptake by upregulating glucose transporters (GLUT1) (Osthus et al., 2000) and it transcriptionally regulates virtually all glycolytic genes (Stine et al., 2015 and references therein). In addition, MYC influences pyruvate kinase mRNA splicing to favor expression of the glycolysis-promoting PKM2 (embryonic pyruvate kinase) isoform (David et al., 2010). In addition to promoting glycolysis, MYC upregulates the expression of PPP genes, which action increases partitioning of glucose carbon to PPP route at the entrance of glycolysis pathway (Morrish et al., 2009; Wang et al., 2011). Among the MYC's glycolytic target genes, for example, hexokinase 2 (HK2) and lactate dehydrogenase A (LDH-A) genes use canonical E-boxes in their promoter regions to recruit MYC (Kim et al., 2004). These genes likely represent the class of regular metabolic targets of MYC, influenced already by low levels of constitutive MYC expression in normally proliferating cells (Figure 1). Recent evidence suggests that some of the MYC influenced target genes with low affinity promoters for MYC encode proteins involved in nutrient transport, glycolysis and hypoxia responses (Lorenzin et al., 2016).

Thus, it is likely that a sub-set of MYC's metabolic target genes reside beyond the classical high-affinity E-box promoter domain and therefore, comprise the group of genes that is only affected by the supraphysiological MYC levels (Figure 1). The projected benefit of increased anabolic metabolism for tumor cells generates selection pressure toward supraphysiological MYC expression but these metabolic transformation-specific signaling circuits also create cancer vulnerabilities for therapeutic intervention (discussed below, Lorenzin et al., 2016).

### Glutaminolytic programs

MYC has a notable role in regulation of glutamine metabolism, and many recent findings elucidating the specific role of MYC in glutaminolytic processes have stimulated broader interest in glutamine as an essential nutrient for cancer cells. Glucose and glutamine are both cells' primary carbon sources for ATP production and biosynthesis and these molecules are usually available in high quantities since glutamine is the most abundant circulating free amino acid in human blood (Mayers and Vander Heiden, 2015). Glutamine is consumed in large quantities in stressed and rapidly dividing cells (such as lymphocytes, enterocytes of the small intestine and cancer cells) and used for both bioenergy generation and as a source of carbon and nitrogen for biosynthesis (Altman et al., 2016). MYC stimulates glutamine uptake through regulation of glutamine transporters (Wise et al., 2008). In the cytosol, glutamine can directly contribute to nucleotide biosynthesis as an obligate nitrogen donor (Lane and Fan, 2015). In the first step of glutaminolysis, glutamine is converted to glutamate (glutamic acid) by action of mitochondria-localized glutaminase (GLS1). MYC promotes this step through transcriptional and posttranslational regulation of GLS1 expression (Gao et al., 2009; Wise and Thompson, 2010; Haikala et al., 2016). Cytosolic glutamate is the primary nitrogen donor for the synthesis of many non-essential amino acids (serine, alanine, aspartate, and ornithine) (Gao et al., 2009; Wise and Thompson, 2010). Glutamate is then further metabolized to α-ketoglutarate (α-KG), which is one of the intermediates of Krebs cycle (a.k.a tricarboxylic acid or TCA or citric acid cycle) (Wise and Thompson, 2010). α-KG is further oxidized in Krebs cycle to generate ATP and to provide carbon skeletons for macromolecule synthesis (i.e., nucleotides, proteins and hexosamines) (DeBerardinis et al., 2007; Wellen et al., 2010; Altman et al., 2016). Several metabolic profiling studies, exploring the fate of glucose- or glutamine-derived carbon in the cells' metabolic processes, have provided evidence that MYC-dependent glutaminolysis stimulates and may indeed fuel Krebs cycle (DeBerardinis et al., 2007; Le et al., 2012; Yuneva et al., 2012). For example, under MYC's influence and low glucose conditions, the glutamine-derived carbons become enriched in the Krebs cycle intermediates fumarate, malate and citrate (Le et al., 2012). Therefore, under the influence of MYC, cells may use glutamine as a key anaplerotic substrate to maintain Krebs cycle-dependent biosynthetic reactions (see below). One of the most striking features of MYC transformed cells is their strict dependence of glutamine for survival (Wise et al., 2008). The glutamine-addiction of MYC transformed cells provides possible therapeutic opportunities and for example, pharmacologic inhibition of GLS1 has been shown to inhibit tumor progression in mouse models of MYC-driven Burkitt's lymphoma or hepatocellular carcinoma (Le et al., 2012; Xiang et al., 2015).

### Glutamine anaplerosis

Otto Warburg originally attributed poor utilization of the mitochondrial oxidative phosphorylation by cancer cells to mitochondrial damage. However, while glycolysis-derived intermediates can importantly contribute to biosynthesis, they cannot make up the whole pool of biosynthetic molecules in the cell, which indicates a role for mitochondrial Krebs cycle in anabolic reactions of the cancer cells (Ochoa-Ruiz and Diaz-Ruiz, 2012). Indeed, emerging evidence suggest both inter—and intratumor heterogeneity as well as metabolic flexibility in the cancer cells' exploitation of glycolysis vs. Krebs cycle for energy production and biosynthesis (Ochoa-Ruiz and Diaz-Ruiz, 2012). Krebs cycle not only produces ATP but also number of metabolites for anabolic reactions. Typical examples of Krebs cycle anabolic precursors include citrate that is used for fatty acid synthesis or oxaloacetate that takes part in gluconeogenesis, amino acid and lipid synthesis (Altman et al., 2016). The enhanced efflux of Krebs cycle intermediates in cancer cells for biosynthetic reactions is to some extent balanced by accelerated glycolysis that boosts the influx of pyruvate to Krebs cycle. However, it is believed that enhanced glycolysis is not alone sufficient to compensate substantial efflux of Krebs cycle intermediates to biosynthesis in rapidly growing and proliferating cancer cells. The additional compensation comes from anaplerotic reactions, which refill the Krebs cycle with metabolites at the discrete steps where they are diverted away from the cycle (efflux) for biosynthesis (Ochoa-Ruiz and Diaz-Ruiz, 2012). For example, the glutamine -derived metabolite α-ketoglutarate (α-KG) is part of Krebs flux, between isocitrate and succinyl-CoA (Altman et al., 2016). The importance of glutamine as an anaplerotic precursor has been long studied in context of physiological responses of muscles or other organs to exercise and starvation (Bowtell and Bruce, 2002; Owen et al., 2002).

As discussed above, MYC's effect on glutaminolysis can fuel the Krebs cycle (Le et al., 2012). The importance of anaplerosis for the metabolism of transformed cells has been addressed for example in glioblastoma cells exhibiting aerobic glycolysis. DeBerardinis et al. showed a rescue of glioblastoma cells from glutamine deprivation-induce death with a cell permeable form of α-KG, which cannot act as an amide donor in nucleotide biosynthesis or as nitrogen source for the synthesis of non-essential amino acids (DeBerardinis et al., 2007). Therefore, the anaplerotic role of glutamine appears to be an important factor in glutamine addiction of transformed cells. However, it appears that MYC-induced diversion of glutamine to support cell growth and proliferation requires more from glutamine than only anaplerosis, since replacement of glutamine with α-ketoglutarate (α-KG) in context of MYC-induced proliferation fails to rescue the cell cycle progression (Wang et al., 2011). Nevertheless, α-KG is sufficient to rescue cells from MYC-induced apoptosis in a model that involves perturbation of RhoA-SRF-dependent GLS1 regulation (Haikala et al., 2016). Thus, it is tempting to speculate that glutamine anaplerosis is critical for protection of cells from MYC-dependent apoptosis whereas increased glutamine utilization, which involves glutamine's contribution to synthesis of nucleotides and non-essential amino acids, is required for cell proliferation and cell growth.

## MYC and AMPK–at the metabolic ambivalence

### Cell viability affairs: consequences of declining ATP levels

As discussed earlier, the necessity of incessantly proliferating cells to shift their metabolic programs toward anabolic reactions occurs at the expense of ATP production. However, an adequate supply of ATP is necessary for normal cell functions and beneath that adequacy cells will die. For example, even a transient drop of cellular ATP levels in HeLa and other tumor cells is sufficient to kill the cells by means of mitochondrial regulated apoptosis (Vander Heiden et al., 1999; Izyumov et al., 2004). Since low ATP level is both a signal of metabolic stress and a state that needs rapid countering actions to restore energy, cells have evolved specific mechanisms to monitor the AMP/ADP:ATP ratio in the cells.

The principal cellular energy sensor is AMP activated kinase (AMPK), which is composed of the catalytic α-subunit and two regulatory subunits β and γ. AMPK has four adenine nucleotide-binding clefts of which two (sites 1 and 3) bind AMP, ADP, or ATP in a competative manner (Hardie, 2014; Zadra et al., 2015). In unstressed or resting cells with high ATP:ADP, these sites are predominantly occupied by ATP but under metabolic stress, the increased levels of AMP and ADP in relation to ATP will lead to progressive replacement of ATP with AMP or ADP in the two AMPK's responsive nucleotide-binding sites. AMPK activity is further regulated via phosphorylation of Ser172 site, which resides in the activation loop of kinase domain. The upstream kinase that phosphorylates this regulatory phosphosite of AMPK is LKB1 (liver kinase B1), which is a tumor suppressor mutated or silenced in various sporadic cancers and in an inherited cancer susceptibility syndrome, Peutz-Jeghers syndrome (Shaw et al., 2004; Shackelford and Shaw, 2009; Hardie, 2015).

### AMPK–integrating catabolic processes to checkpoints of cell cycle and death

AMPK activation coordinates number of metabolic signaling pathways with the general purpose of switching on ATP generating catabolic pathways, while simultaneously switching off ATP consuming biosynthetic pathways. Some of these pathways will be discussed in more detail below but the principal impacts of AMPK activation on the catabolic pathways include stimulation of glucose uptake, glycolysis, fatty acid uptake, fatty acid oxidation, mitochondrial biogenesis and autophagy. AMPK activates the main mitochondrial biogenesis inducer PGC-1α, which then activates sequence of events via NRF-1 and NRF-2 transcription factors leading to increased production of mitochondrial enzymes as well as transcription and replication of mitochondrial DNA (Jornayvaz and Shulman, 2010). The AMPK-mediated mitochondrial biogenesis has been implicated in maintenance of energy homeostasis and cancer cell survival (Chaube et al., 2015). The invariably AMPK-inhibited anabolic pathways include inhibition of fatty acid synthesis, lipogenic enzymes, triglyceride and cholesterol synthesis, transcription of gluconeogenic enzymes, glycogen synthesis, protein synthesis via mTOR, and ribosomal RNA synthesis. The AMPK-mediated catabolic effects and associated target proteins are discussed here only in the light of examples as AMPK pathways are thoroughly covered in many recent excellent reviews (Mihaylova and Shaw, 2011; Hardie, 2014; Hardie et al., 2016).

The AMPK-induced ubiquitous negative impact on anabolic metabolism suppresses cell growth and proliferation. AMPK inhibits growth at least partly via AMPK-mediated phosphorylation of TSC2 and raptor, which events inhibit mTORC1 activity (Shackelford and Shaw, 2009). The inhibitory effects of AMPK on cell proliferation additionally include for example, suppression of BRAF and Hippo pathways mediator YAP (Zadra et al., 2015 and references therein). AMPK has also been considered as a mediator of metabolic G1/S checkpoint, which is triggered by glucose deprivation. Lack of glucose activates AMPK, which directly phosphorylates the N-terminal Ser15 of p53, leading to initiation of p53-dependent cell-cycle arrest or if the AMPK activity remains persistent, to cellular senescence (Jones et al., 2005).

In addition to quiescence, metabolic activation of the AMPK-p53 axis can have more grave consequences to the cells. For example, glucose-deprivation induces AMPK and p53-dependent cell death in thymocytes and in human bone osteosarcoma U2OS cells (Okoshi et al., 2008). In this study, the increased AMPK activity associated with p53 phosphorylation at Ser46, which site has been previously implicated in apoptotic function of p53 (Oda et al., 2000; Okoshi et al., 2008). Furthermore, ATP depletion during neuronal excitotoxicity (bioenergetic failure through glutamate receptor overactivation) triggers activation of AMPK and if this activation is prolonged, it will eventually lead to apoptosis through the action of pro-apoptotic Bcl-2 family member BIM (Concannon et al., 2010). In this system, the AMPK-induced cell death involves AMPK-dependent inhibition of AKT kinase, which leads to two distinct phosphorylation events that target the transcription factor FOXO3, which is followed by FOXO3-induced upregulation of BIM (Brunet et al., 1999; Davila et al., 2012). Sustained activation of AMPK has also been linked to c-Jun N-terminal kinase (JNK) and caspase-3-mediated apoptosis in liver cells (Meisse et al., 2002).

### MYC and metabolic stress-induced cell death pathway: MYC triggers AMPK-dependent activation of P53

Given the opposing roles of MYC and AMPK pathways in regulation of cell metabolism, it can be anticipated that cells with both pathways simultaneously active will endure a significant amount of stress with potentially dire consequences. Several studies, including our own, have shown that an acute activation of MYC induces or contributes to depletion of the cellular ATP reservoirs and leads to concomitant activation of AMPK (Liu et al., 2012; Nieminen et al., 2013). In these circumstances AMPK strongly phosphorylates p53 at N-terminal serine 15, which modification is known to liberate p53 from MDM2-dependent inhibition and consequently, this leads to stabilization of p53 (Kruse and Gu, 2008).

However, from this point on, the nature of the p53 stabilizing signal appears to influence the subcellular locale where p53 accumulates. In our study, we observed that while administration of chemotherapeutic agents to mammary epithelial MCF10A cells (without active MYC) led to nuclear accumulation of p53, the activation of MYC in the same cells re-routed p53 to interact with BAK and BCL-X_L_ in the mitochondria. These events led to conformational activation of BAK, which associates with higher sensitivity of the cells to apoptosis (Nieminen et al., 2013). These findings were consistent with earlier studies suggesting a role for cytosolic p53 in direct physical regulation of the mitochondrial BCL-2 family members (Green and Kroemer, 2009; Vaseva and Moll, 2009). Importantly, whilst MYC-AMPK-p53 axis induced the N-terminal exposure of BAK, the protein did not seem to dissociate from the inhibitory complex with anti-apoptotic BCL-X_L_. These cells with conformationally active BAK did not outright commit suicide by apoptosis but were primed for death induction (Nieminen et al., 2007, 2013).

From these findings we formulated the hypothesis that non-transformed cells have the ability to deal with declining ATP levels because of AMPK-p53-mediated checkpoint control mechanism (Jones et al., 2005). This metabolic checkpoint mechanism arrests the cell cycle before the ATP levels drop too low and thus, endows cells time to recover their ATP reservoirs via catabolic pathways. On the contrary, the cancerous cells with deregulated MYC expression will not be able to downregulate MYC, nor exit from the cell cycle and nor switch off the anabolic pathways. Therefore, these cells will continuously cycle under low ATP levels and with active AMPK, leading to progressive accumulation of p53 in the mitochondria and gradually increasing sensitivity to apoptosis (Nieminen et al., 2013). Such mechanism could have evolved to fulfill the role of an intrinsic tumor suppressor mechanism, which limits the proliferation of the cells with out-of-control cell cycle control (Lowe et al., 2004).

### Complex relationship between MYC and AMPK in transformation and tumorigenesis

The findings discussed so far have highlighted AMPK as a metabolic checkpoint protein and a potential tumor suppressor protein, which claim is supported by large number of studies exposing the anti-growth, anti-proliferative and anti-survival actions of activated AMPK. However, it is now clear that the role of AMPK in cancer is more complex and highly contextual (Liang and Mills, 2013; Zadra et al., 2015). Nevertheless, the mechanistic findings related to AMPK's potential anti-cancer actions have suggested that AMPK activating compounds, some of which have been used for decades to treat metabolic disorders, could be re-profiled for treatment of cancer (Fogarty and Hardie, 2010). These therapeutic initiatives are substantiated by the findings made over 10 years ago that patients with type 2 diabetes and treated with metformin, which is an indirect activator of AMPK, had significant lower risk of developing cancer than patients on other medications (Evans et al., 2005; Bowker et al., 2006).

Specifically, the evidence for a tumor suppressor role of AMPK in context of MYC expression comes from the studies of Faubert et al, demonstrating that inactivation of the catalytic α1-subunit of AMPK accelerates MYC-driven lymphomagenesis (Faubert et al., 2013). In these experiments, depletion of AMPK also favored aerobic glycolysis. For example, the AMPK-less tumor cells showed increased glucose consumption, increased lactate production and upregulation of transcription factor HIF-1α and its glycolytic downstream targets (LDH-A, PDK1, and ALDA). Furthermore, the tumors exhibited increased flux of glucose carbons into lipids, indicating transition to anabolic metabolism. Interestingly, the metabolic shift was reversed by silencing of HIF-1α, suggesting that AMPK and HIF-1α have opposing roles in the control of tumor metabolism.

Liver kinase B1 (LKB1) positively regulates the activity of at least 14 AMPK related downstream kinases (AMPK and ARKs; Katajisto et al., 2007), so no direct equivalence between loss of LKB1 and loss of AMPK can be drawn but it is intriguing to note that loss of LKB1 (and inferring from that, loss of AMPK activity) dramatically promotes the oncogenic properties of MYC in 3D culture system as well as MYC-dependent tumorigenesis in mouse mammary gland (Partanen et al., 2007, 2012). Loss of LKB1 has also been shown to support growth-promoting metabolism through mTORC1 hyperactivation and reactive oxygen species-(ROS) dependent activation of HIF-1α (Shackelford et al., 2009; Faubert et al., 2014).

Genetic mutations in AMPK are not frequent in human cancer, although both point mutations and gene amplifications have been observed (Liang and Mills, 2013). One study in breast cancer has reported that AMPK is downregulated in majority of examined cases and that reduced phospho-AMPK signal correlates with breast cancer aggressiveness (Hadad et al., 2009).

Contrary to the suggested role of AMPK as a tumor suppressor, there is also evidence that depletion of LKB1 or AMPK, and consequent loss of bioenergetic control, hypersensitizes cells to apoptosis and renders cells resistant to transformation (Shaw et al., 2004; Liang and Mills, 2013). In context of MYC expression, Liu et al., demonstrated synthetic lethal interaction between MYC and silencing of AMPK-related kinase 5 (ARK5), which is upstream regulator of AMPK (Liu et al., 2012). ARK5 was found to be essential for MYC-driven expression of the mitochondrial respiratory chain proteins and glutamine metabolism (Liu et al., 2012).

The current findings, which have provided support for both tumor beneficial and pestilent AMPK functions, are perhaps not contradictory if we consider the basal and high AMPK activity as separate entities. We postulate that the basal level of AMPK activity, which is important for bioenergetic homeostasis of proliferation-active cells, is likely to be beneficial for tumor growth at large whereas a high or prolonged catabolic AMPK activity generates an imminent conflict with the expression of oncogenes, such as MYC, which drive strongly anabolic growth promoting pathways. The scenario, if true, would be highly interesting from the therapeutic standpoint since AMPK activating drugs could make the metabolic conflict worse and promote selectively cell death in MYC transformed cells. Then, if the tumor cells evolve to survive apoptosis through inactivation of AMPK, this would lead to another type of apoptotic sensitivity in escapee tumor cells due to lack of proper bioenergetic control systems.

## Future prospects

MYC has been for decades one of the most intensively studied oncoprotein, and while the protein itself is not targetable by traditional pharmacological approaches, the MYC-dependent pathways have formed a targetable domain for variety of synthetic lethal approaches. To mention few of recently identified plethora of MYC-dependent metabolic vulnerabilities, MYC is synthetic lethal with losses engineered to glucose metabolism genes, nucleotide metabolism genes, glutamine/glutamate transporters, or to genes encoding glycolysis or lipogenesis enzymes (reviewed in Stine et al., 2015). In addition, pharmacological “tool compound” inhibitors of LDH-A, GLS or lactate exporter MCT1 have been shown to inhibit MYC-dependent tumorigenesis in mouse models of cancer (Le et al., 2010, 2012; Wang et al., 2010; Doherty et al., 2014).

However, the general problem with any type of signaling intercepting strategy is that tumor cells quickly adapt to the interception and evolve to use alternative signaling pathways to restore the inhibited signaling capacity. The redundancy of kinase pathways, rendering cells resistant to clinical EGFR-tyrosine kinase inhibitors is a well-known example (Sun and Bernards, 2014). Therefore, on one hand, it can be anticipated that the broad repertoire of drugs for treating metabolic disorders will facilitate new drug development and drug repositioning initiatives aiming to exploit metabolic cancer vulnerabilities. On the other hand, it is also a serious concern that the enormous complexity and highly adaptive nature of metabolic networks will provide many escape routes for metabolically targeted tumors, which will eventually lead to therapy resistance.

We propose, not as a fact but as an incentive for future studies, that the dual role of AMPK as an essential guardian of cellular bioenergetic homeostasis and a formidable driver of catabolic metabolism may set AMPK apart as a potentially non-redundant cancer metabolic target. As discussed above, high or persistent AMPK activity promotes induction of transient or permanent cell cycle arrest or apoptosis. Therefore, AMPK activating compounds, such as biguanides metformin and phenformin or an allosteric activator A-769662 (Cool et al., 2006), could offer pharmacological strategies to establish synthetic lethality with MYC. Tumor cells could attempt escape from the synthetic lethality with MYC by downregulating AMPK activity, which event, however, would endanger tumor cells' lives due to loss of bioenergetic homeostasis.

Could tumor cells potentially evade AMPK's anti-proliferative or anti-survival functions by mutating p53? As discussed above, both the glucose stress- and MYC-induced AMPK activity induces p53 phosphorylation and stabilization, which promotes cell cycle arrest, premature senescence or apoptosis (Jones et al., 2005; Okoshi et al., 2008; Nieminen et al., 2013). Therefore, it is clear that AMPK-p53 signaling axis is tightly coupled to anti-proliferative or anti-survival AMPK functions. It is also notable that metformin- or AICAR- (AMP analog that stimulates AMPK activity)-induced cell cycle arrest in G1-phase associates with increased phosphorylation of p53 at Ser15 (Wonsey et al., 2002; Morrish et al., 2010; Priolo et al., 2014). Nevertheless, the question about the requirement of wild-type p53 specifically for AMPK's pro-apoptotic and anti-tumorigenic effects is unresolved. There is *in vivo* evidence that metformin (or AICAR) exerts apoptotic effects in p53-deficient, but not in the wild type p53 xenografts (Buzzai et al., 2007). However, another study suggests that wild type p53 is required for the antitumor effects of metformin (Li et al., 2015). It is important to note that the missense mutated p53 proteins, which are typically expressed in cancer, do have well-established gain of function, transcription-independent and mitochondrial apoptosis associated functions although the specific impacts of missense mutations on the p53 function, including capacity to mediate cell death, is debated (Vaseva and Moll, 2009; Freed-Pastor and Prives, 2012). Therefore, the question about the role of wild type and mutant p53 in mediating the metabolic stress and AMPK-dependent cell death warrants further studies.

**Figure 3:**
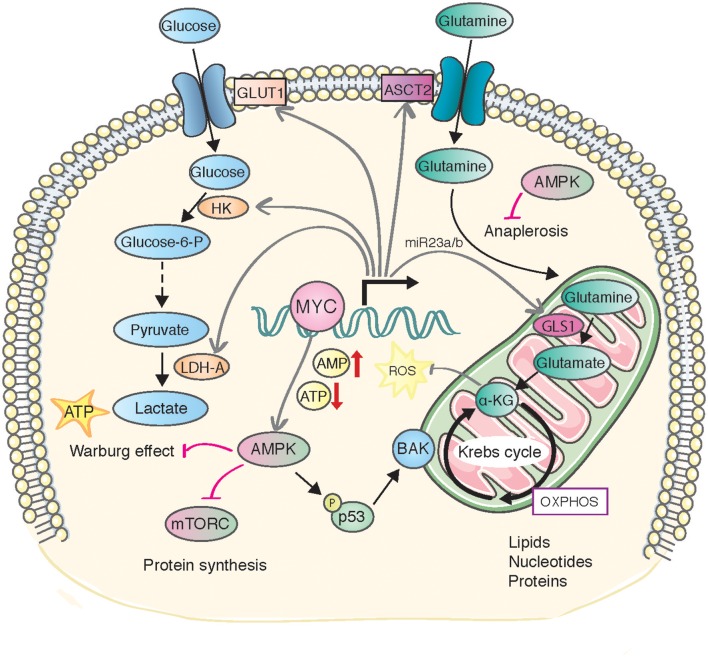
**A model of metabolic stress and consequences caused by MYC-induced AMPK activity**. MYC-induced metabolic transformation leads to declined ATP levels and enhanced AMPK activity. AMPK activity predominantly stimulates catabolic reactions, generating conflicting signals with the MYC-induced anabolic pathways (depicted in the figure, see text for details). The metabolic stress is directly or indirectly sensed by p53, which can contextually induce permanent cell cycle arrest (senescence) or sensitize cells to apoptosis.

It is tempting to speculate that MYC-induced anabolic reactions are highly incompatible with a persistently activated catabolic AMPK function, creating an unresolvable metabolic stress that exerts anti-proliferative or anti-survival effects independently of p53 (Figure 3). For example, MYC-driven tumor cells are highly dependent on ribosome biogenesis and protein synthesis, requiring a collaboration between MYC and mTOR signaling to satisfy the increased biosynthetic needs (van Riggelen et al., 2010; Pourdehnad et al., 2013). Persistent AMPK activity directly antagonizes mTOR-driven protein synthesis (Bolster et al., 2002; Inoki et al., 2003; Dreyer et al., 2006) and such catabolic program could create a synthetic lethal crisis in MYC expressing cells. Earlier studies have suggested a highly context-dependent role for mTOR in regulating apoptosis (Castedo et al., 2002), and it remains for future studies to resolve how mixed input signals to mTOR pathway might affect to cell viability. Several reports have suggested that metformin and phenformin downmodulate MYC levels in prostate and breast cancer cells (Blandino et al., 2012 PMID: 22643892, Akinyeke et al., 2013). This modulation has been suggested to occur via upregulation of mir-33a, which targets MYC (Blandino et al., 2012). However, the exact role of AMPK in this pathway has not been demonstrated.

One obvious scene of interest for future studies is the antagonistic relationship of AMPK and MYC in anaplerosis and how that will influence cell viability. Glutamine-deprivation induced apoptosis of tumor cells and MYC-transformed cells can be rescued by addition of exogenous alpha-ketoglutarate (α-KG) to the cells, suggesting that the anaplerotic flux of glutamine into the Krebs cycle is a critical survival mechanism (DeBerardinis et al., 2007; Haikala et al., 2016). Besides the Krebs cycle promoting function, glutamine anaplerosis and α-KG have a role in protecting cells against reactive oxygen species (ROS), constituting an additional glutamine related pro-survival mechanism (Fedotcheva et al., 2006; Mailloux et al., 2007; Niemiec et al., 2011). Indirect AMPK activator metformin was recently shown to decrease the flow of glucose- and glutamine-derived carbon into the Krebs cycle, leading to reduced citrate production and lipid synthesis (Griss et al., 2015). Such antagonizing effects of AMPK activity on glutamine utilization could be selectively harmful for addicted tumor cells and not such for normal cells. Further clarification of the role of anaplerotic mechanisms as potential life-lines of metabolically transformed tumor cells may not only new shed light to intricacies of cancer cell metabolism but also pave way for new effective cancer therapies.

## Author contributions

HH, JK, and JA wrote the paper. HH (and JA) prepared the figures.

## Funding

This work was funded by the Academy of Finland, TEKES, and Finnish Cancer Organizations. HH and JA were funded by Integrative Life Sciences (ILS) doctoral program. HH was funded by Emil Aaltonen foundation, Inkeri and Mauri Vänskä Foundation and Biomedicum Helsinki foundation.

### Conflict of interest statement

The authors declare that the research was conducted in the absence of any commercial or financial relationships that could be construed as a potential conflict of interest.
